# Gleason Grade Group 4 prostate biopsy with no cancer seen on final pathology in the magnetic resonance imaging and Prostate Specific Membrane Antigen‐Positron Emission Tomography era

**DOI:** 10.1002/iju5.12614

**Published:** 2023-08-11

**Authors:** Nikhile Mookerji, Rohan Mittal, Amaris Hui, Tyler Pfanner, Stacey Broomfield, Luke Dean, Benjamin Adam, Christopher Fung, Alexander Tamm, Adam Kinnaird

**Affiliations:** ^1^ Division of Urology, Department of Surgery University of Alberta Edmonton Alberta Canada; ^2^ Department of Pathology University of Alberta Edmonton Alberta Canada; ^3^ Department of Radiology & Diagnostic Imaging University of Alberta Edmonton Alberta Canada; ^4^ Alberta Prostate Cancer Research Initiative (APCaRI) Edmonton Alberta Canada; ^5^ Cancer Research Institute of Northern Alberta (CRINA) Edmonton Alberta Canada; ^6^ Alberta Centre for Urologic Research and Excellence (ACURE) Edmonton Alberta Canada; ^7^ Department of Oncology University of Alberta Edmonton Alberta Canada

**Keywords:** multiparametric MRI, prostate cancer, PSMA PET, vanishing cancer

## Abstract

**Introduction:**

The absence of prostate cancer on final surgical pathology after biopsy‐proven prostate cancer is a rare finding.

**Case presentation:**

Case of pT0 prostate cancer following Gleason Grade Group 4 in 1 out of 12 cores from a transrectal ultrasound‐guided biopsy in a man who underwent both magnetic resonance imaging and ^18^F‐PSMA‐1007 Positron Emission Tomography prior to radical prostatectomy.

**Conclusion:**

pT0 prostate cancer is rare. The use of novel imaging modalities may help in the workup of prostate cancer.

**Abbreviations & Acronyms**
CT: computed tomography
GGG 4: Gleason Grade Group 4
H&E: hematoxylin and eosin
HGPIN: high‐grade prostatic intraepithelial neoplasia
mpMRI: multiparametric magnetic resonance imaging
MRI: magnetic resonance imaging
PET: Positron Emission Tomography
PSA: prostate specific antigen
PSMA: Prostate Specific Membrane Antigen
RP: robotic prostatectomy
TRUS: transrectal ultrasound‐guided

**Keynote message**

This is an uncommon case of high‐risk prostate cancer found on biopsy with no cancer found on final surgical pathology. The patient had PSMA PET and MRI prior to surgery to help with locoregional staging. This case demonstrates the utility and limitations of new imaging techniques in the staging of prostate cancer and the importance of combining both imaging and biopsy in the diagnosis of prostate cancer.

## Introduction

Pathological T0 prostate cancer after radical prostatectomy is an uncommon and unpredictable finding. Also called *vanishing prostate cancer*, this phenomenon is of particular importance for patients who have not received prior hormonal therapy given the medicolegal implications.^1^ Furthermore, the advent of new imaging modalities pre‐operatively may help clinicians prevent this from happening.

## Case presentation

A 63‐year‐old male with a past medical history of benign prostatic hyperplasia and hypertension presented with an elevated PSA at a community Urologist's office in June 2021. He had minimal lower urinary tract symptoms at presentation with nocturia and daytime frequency. He had a benign feeling, enlarged prostate on digital rectal examination. He had no family history of prostate cancer. His PSA was 7.8 (PSA density of 0.13 ng/mL^2^) prior to his biopsy in June 2021. He had prior PSA 5.9 and 4.4 earlier in 2021 (April and March respectively).

He underwent a TRUS prostate biopsy in July 2021 that showed a focus of ductal adenocarcinoma in 1 of 12 cores and involving less than 5% of the biopsy tissue. This was designated GGG 4, high‐risk prostate cancer. He did not experience any post‐procedural complications. Following histological diagnosis, the patient was offered radical prostatectomy (both open and robotic) alongside consultation with radiation therapy. The patient elected for robotic radical prostatectomy (RP) at our tertiary care center.

The patient had conventional imaging to complete his workup following his biopsy. He had a technetium‐99 methylene diphosphonate whole‐body bone scintigraphy that did not identify any bony metastases. This was followed with mpMRI in September 2021 which identified no suspicious measurable abnormality Prostate Imaging Reporting and Database System (PIRADS 2).

**Fig. 1:**
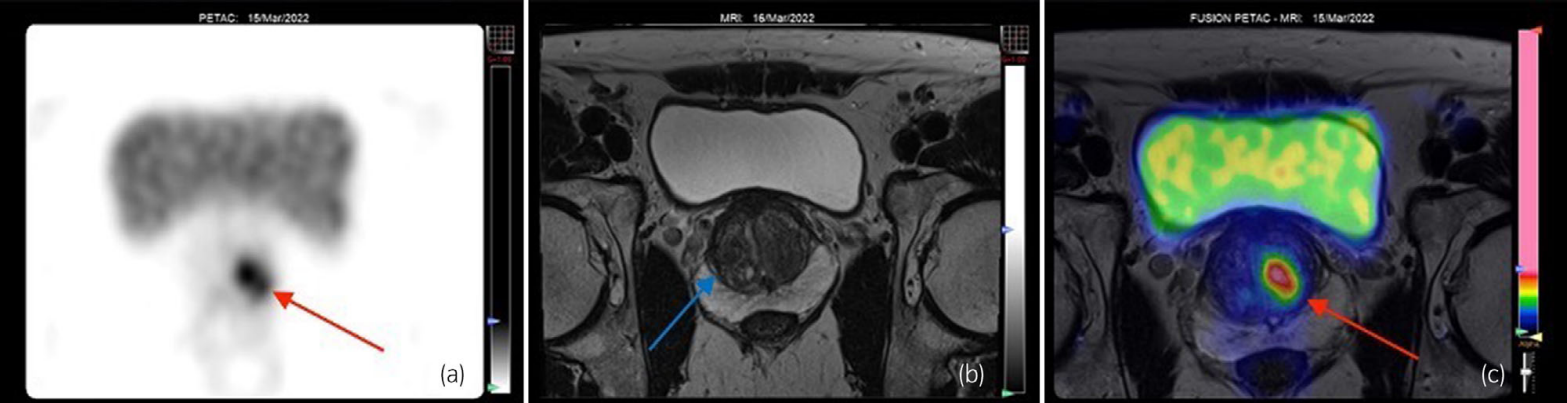
(a) 18F‐PSMA‐1007 PET scan showing left prostatic base activity (red arrow) and none on the right. (b) mpMRI was reported to be PIRADS 2 (blue arrow directed at right prostatic bed where GGG 4 biopsy was taken from). (c) Offline PSMA PET MRI fusion using semi‐automated fusion software identifying increased activity on left base (red arrow).

**Fig. 2:**
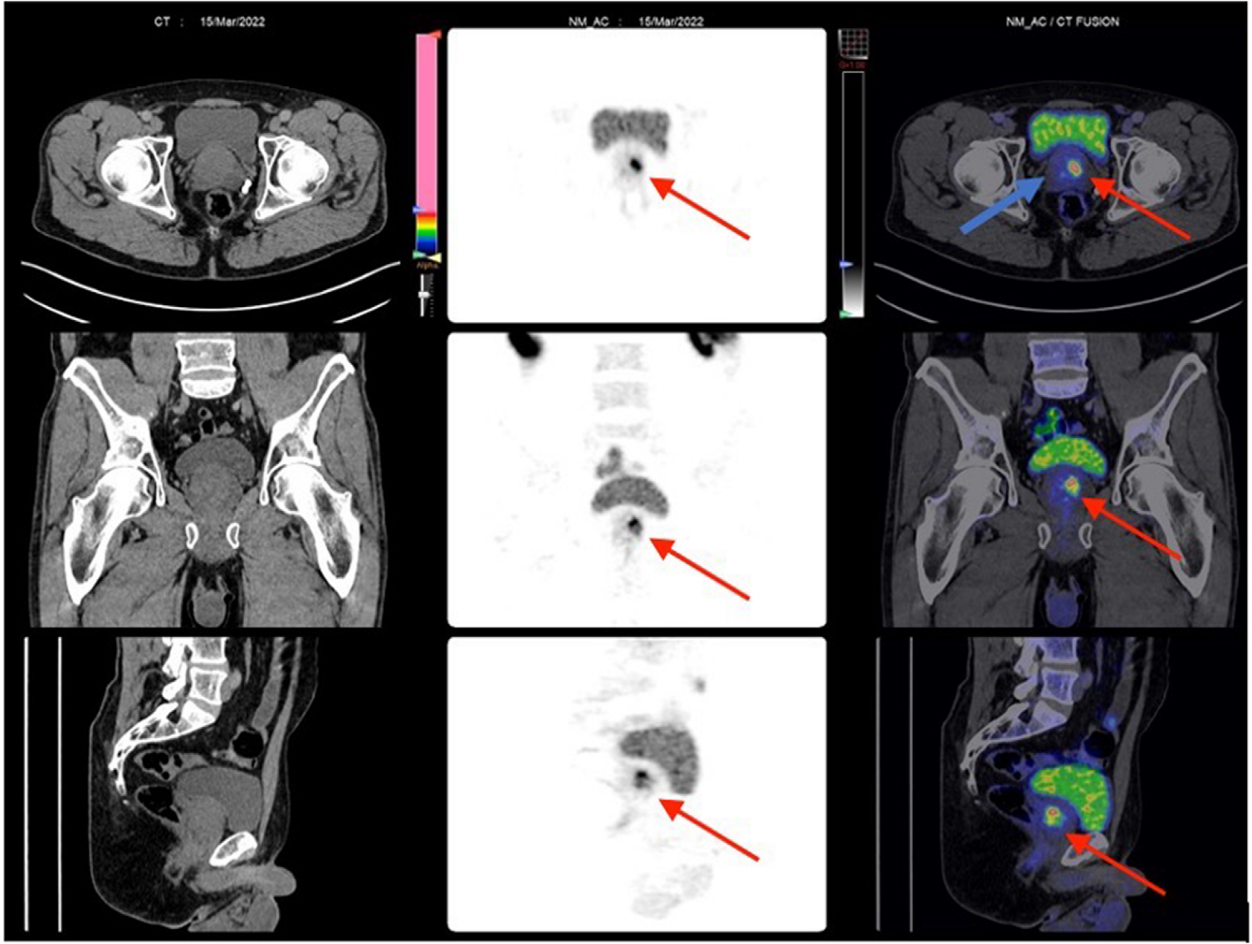
PSMA PET CT. Red arrows identify areas of the prostate (left prostatic base) that had increased uptake. Right base where the TRUS biopsy had been positive does not show any PSMA uptake (blue arrow).

Due to the COVID‐19 pandemic, the patient's surgery had been delayed. He had in the interim enrolled in a clinical trial at our University (NCT05141760) and underwent a Prostate Specific Membrane Antigen Positron Emission Tomography (^18^F‐PSMA‐1007 PET) scan alongside a repeat mpMRI in March 2022. His MRI once again showed no measurable abnormality (PIRADS 2) (Fig. 1). The PSMA PET showed a unifocal organ‐confined lesion in the left base/mid cavity (Figs 1, 2). The lesion had a PSMA expression score of 1, meaning this was equal to or above the blood pool but lower than the spleen. The PSMA‐RADS score, which describes anatomic abnormality, was reported as a score of 4 – a lesion that would be typical for prostate cancer but without a definitive anatomic abnormality. There was no focal abnormal PSMA uptake at the site of biopsy‐proven prostate adenocarcinoma.

The patient underwent an uncomplicated non‐nerve sparring RP in April 2022 and had a typical course in the hospital, being discharged on postoperative day 1. He had a Jackson‐Pratt drain placed in the prostatic bed at the time of the OR that was removed prior to discharge.

The patient's final pathology revealed no invasive carcinoma. Towards the prostatic base, in a similar location to the focus of carcinoma in the biopsy, only occasional foci of HGPIN were identified. This was confirmed by a retained basal cell layer with positive staining with High Molecular Weight Keratin (HMWK) (Fig. 4). Postoperatively, the patient's PSA remained undetectable at 1‐year postop.

## Discussion

The case of vanishing prostate cancer or pT0 among patients who have had prior histologically proven prostate cancer is reported at less than 2%.^1^ For those individuals who have not received pre‐operative neoadjuvant hormonal therapy, the rates are reported to be lower than 1%.^2^ Possible explanations described in the literature include errors in the pathological review of RP or biopsy specimens, mislabeling lab error, or biopsy removing all of the prostate cancer from the organ.^2^

During preparation of the prostate specimen after a prostatectomy, slices of tissue are prepared for pathological review with a microtome. There is a small chance the focus of invasive carcinoma can be lost in this process; however, this explanation is difficult to conclude with any level of certainty in our case.

The management of prostate cancer as a disease process continues to evolve with new imaging and diagnostic modalities that are improving our clinical ability to diagnose clinically significant prostate cancer. Both the Canadian Urological Association and American Urological Association prostate cancer guidelines have been updated in the past few years endorsing the use of mpMRI prior to TRUS biopsy.^3^, ^4^

The case we present here is an unusual case of pT0 prostate cancer following high‐grade GGG 4 prostate cancer in a single core from TRUS biopsy with a negative preoperative MRI. Furthermore, this is a case of particular interest as the patient had an ^18^F‐PSMA‐1007 PET scan, a state‐of‐the‐art imaging modality, being studied for its potential use in prostate cancer. Interestingly, while both the MRI and PET scans suggested an absence of cancer on the ipsilateral side of the positive biopsy, the PET scan was falsely positive for a tumor on the contralateral side. Importantly, this patient's MRI or PET was not completed prior to the biopsy, these were done after the patient had already been diagnosed with prostate cancer and was awaiting his surgery.

A case series from Meissner *et al*. (2021) suggest a possible biopsy‐free diagnostic pathway for patients with concordant mpMRI and PSMA‐PET lesions who can go direct to therapy.^5^ Although our case had discordant imaging, it does emphasize the need for larger studies to help clarify the role of these novel imaging technologies in the treatment of prostate cancer.

**Fig. 3:**
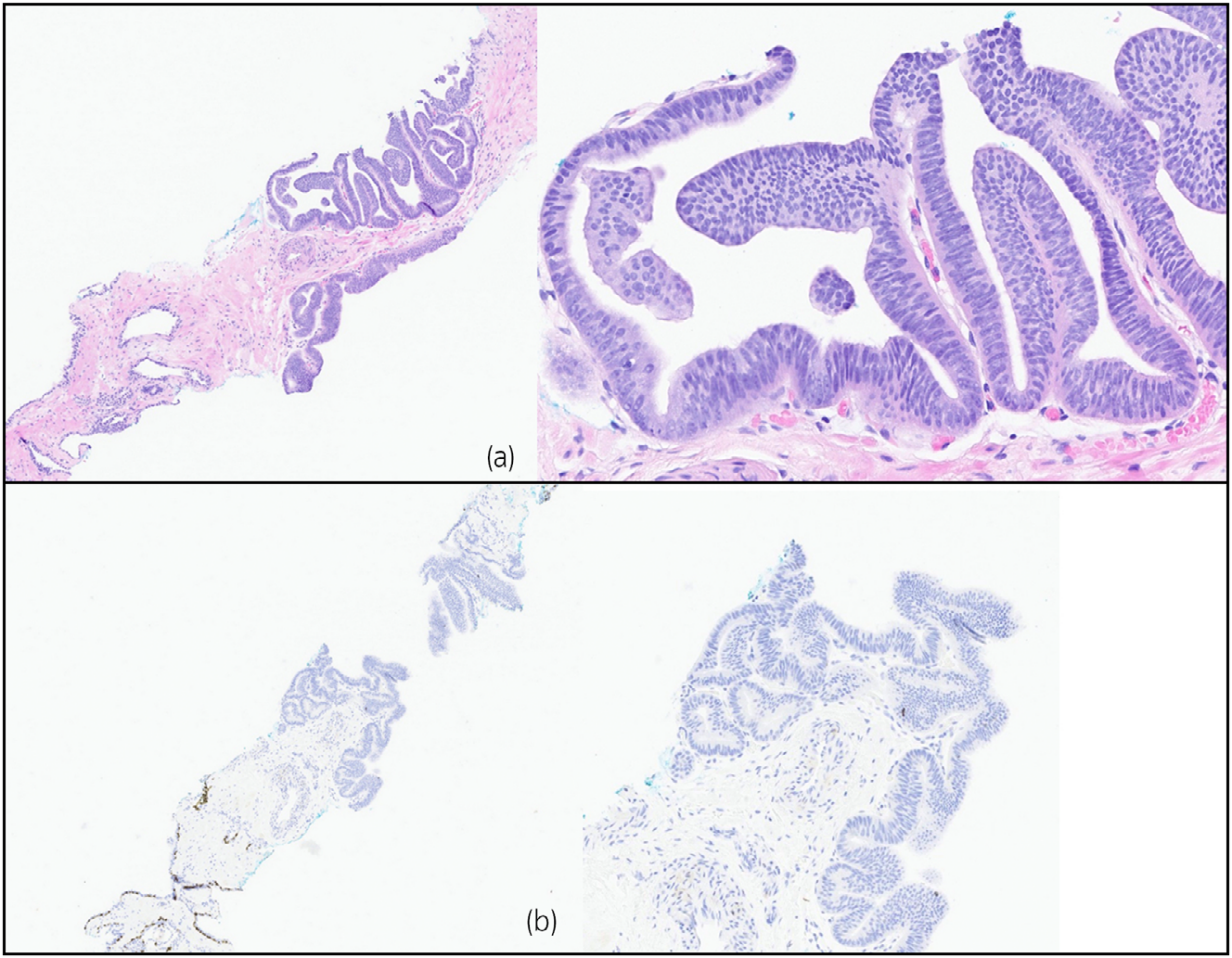
(a) 4× and 20× zoom H&E stain images demonstrating ductal carcinoma in the biopsy sample of right base (b) 4× and 10× zoom immunohistochemistry images demonstrating loss of basal cells in the atypical area consistent with ductal carcinoma.

**Fig. 4:**
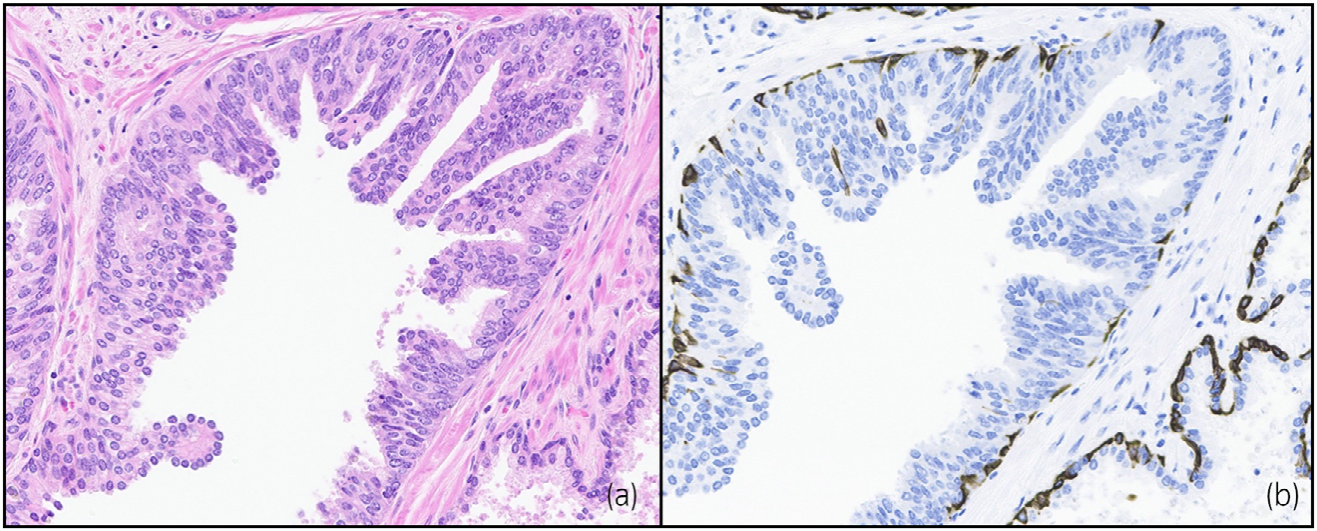
(a) H&E stain demonstrating HGPIN in the final surgical pathology sample of right base (b) HMWK stain of right base on final surgical prostate specimen demonstrating a retained basal cell layer staining and thus diagnosed with HGPIN.

In our case, there were similarities between the TRUS specimen and the final pathology specimen. The right base had similar pathological characteristics except for one critical difference – the absence of the basal cell layer around malignant glands (Figs 3, 4). These suspicious areas in the final pathology specimen were reviewed by multiple genitourinary pathologists who agreed that the basal cell layer was sufficiently retained such that a diagnosis of invasive carcinoma could not be rendered. Finally, we also included additional immunohistochemical staining in Figure S1 which were also negative for malignancy.

## Conclusion

Overall, this case highlights a rare finding of pathological T0 prostate cancer. A clinician should be careful in using novel imaging techniques to navigate the management of prostate cancer. As demonstrated in this paper, PSMA PET can provide a false positive and should be used with caution. There is a role for PSMA PET and MRI in the locoregional staging of prostate cancer, however, TRUS biopsy remains the gold standard for the diagnosis of prostate cancer. The use of MRI and PSMA PET will likely increase over the next decade and understanding their advantages and limitations will be critical.

## Author contributions

Nikhile Mookerji: Investigation; writing – original draft; writing – review and editing. Rohan Mittal: Writing – review and editing. Amaris Hui: Writing – review and editing. Tyler Pfanner: Writing – review and editing. Stacey Broomfield: Writing – review and editing. Luke Dean: Writing – review and editing. Benjamin Adam: Writing – review and editing. Christopher Fung: Writing – review and editing. Alexander Tamm: Supervision; writing – review and editing. Adam Kinnaird: Supervision; writing – review and editing.

## Conflict of interest

There are no conflicts of interest.

## Approval of the research protocol by an Institutional Reviewer Board

This patient was part of a clinical trial. The clinical trial had which did have ethics approval at our institution—IRB—HREBA.CC‐21‐0073.

## Informed consent

Not applicable.

## Registry and the Registration No. of the study/trial

NCT05141760.

## Supporting information


**Fig. S1.** CK 20, CK 7, P504S, NKX3.1, and AR stains at 2.5× zoom that do not demonstrate malignancy.
