# Supplementary figures and images for: Gleason Grade Group 4 prostate biopsy with no cancer seen on final pathology in the magnetic resonance imaging and Prostate Specific Membrane Antigen‐Positron Emission Tomography era
Source: IJU Case Rep. 2023 Aug 11;6(6):337–40. doi: 10.1002/iju5.12614 (PMC10622191; doi:10.1002/iju5.12614)

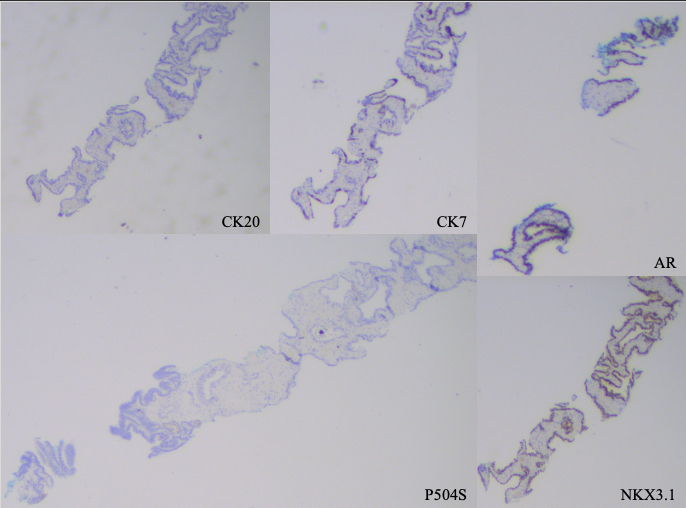

Supplement: Supplementary file 1 — Fig. S1. CK 20, CK 7, P504S, NKX3.1, and AR stains at 2.5× zoom that do not demonstrate malignancy. [file IJU5-6-337-s001.jpg]
